# Exploring the origins of decreased sound tolerance in tinnitus patients

**DOI:** 10.3389/fneur.2023.1273705

**Published:** 2023-11-06

**Authors:** Eun Hye Kim, Seung-Ho Shin, Sung Wan Byun, Ho Yun Lee

**Affiliations:** Department of Otorhinolaryngology-Head and Neck Surgery, Ewha Womans University School of Medicine, Seoul, Republic of Korea

**Keywords:** tinnitus, sound intolerance, hyperacusis, auditory brainstem evoked potentials (ABR), otoacoustic emission (OAE)

## Abstract

This study aimed to confirm the characteristics of auditory function alterations in tinnitus patients with concomitant decreased sound tolerance (ST) and provide insights for developing tailored therapeutic approaches. A retrospective analysis was conducted on patient records from a tertiary university hospital's tinnitus clinic between March 2020 and June 2023. Demographic attributes and audiological profiles were reviewed. Patients were categorized into Group 1 if loudness discomfort level test outcomes were 77 dB or below, measured using an average of frequencies from 250 Hz to 8 kHz. The remaining patients were allocated to Group 2. Among the 434 tinnitus patients, 115 (26.5%) demonstrated decreased ST and were classified as Group 1. This group exhibited higher DPOAE amplitudes (*p* < 0.001), shortened latency, and decreased threshold of ABR wave V bilaterally (*p* < 0.05). No significant disparities were observed in gender, age, tinnitus handicap inventory, visual analog scale, and pure-tone audiometry results except subjective hyperacusis. Binary logistic regression analysis utilizing the forward conditional method revealed that the difference between groups was independently linked to DPOAE response at 7,277 Hz on the left side [B = 0.093, *p* < 0.001, EXP(B) = 1.07, 95% CI = 1.044–1.153]. Increased DPOAE amplitude and shorter and decreased ABR wave V in tinnitus patients with decreased ST might suggest a possible association with lesions in or around the superior olivary complex or higher central auditory pathway, potentially linked to the inhibition of medial olivocochlear efferents.

## 1. Introduction

Tinnitus, defined as the conscious perception of sound or noise without external auditory stimuli (1), has a reported global prevalence of 14.4% (4.1–37.2%) in adults, with incidence rising with age (2). Tinnitus generation is typically attributed to bottom-up and/or top-down mechanisms. Anatomically, the medial geniculate body (MGB) accepts afferent input from the inferior colliculus and relays information to primary or secondary auditory cortexes (3). Furthermore, the MGB interacts with limbic structures, including the amygdala, nucleus accumbens, and hippocampus, and is also influenced by inhibitory inputs from the primary auditory cortex and limbic system through thalamic reticular nuclei. Alterations in the MGB and its associated connections can potentially influence tinnitus perception. Additionally, the interplay of the salience network, default mode network, and central executive network may account for the emotional and functional distress reported by tinnitus patients (4).

In the scenario of decreased peripheral input, tinnitus is often associated with auditory irregularities. Prior research has underscored decreased sound tolerance (ST) as a common attribute in tinnitus patients with normal hearing, alongside extended high-frequency hearing loss (EHFHL), abnormal electrocochleography (ECoG), and shifts in distortion product otoacoustic emissions (DPOAE) and auditory brainstem responses (ABR) (5).

Decreased ST can be evaluated via the loudness discomfort level (LDL) test or the Khalfa hyperacusis questionnaire (HQ). In LDL-based audiological assessments, various criteria for hyperacusis or auditory hypersensitivity are employed, including a dynamic range below 60 dB, LDLs of 90 dB or less at two or more frequencies, LDLs <90 dB HL between 500–8 kHz, and 70 dB at 250 Hz, among others (6). Some studies have noted a correlation between questionnaire findings and audiological tests when a mean of the lowest LDLs ≤ 77 dB HL and an HQ score ≥22 are utilized for diagnosing hyperacusis (7). However, the LDL assessment can be subjective and potentially uncomfortable for patients. Conversely, auditory evoked responses such as DPOAE, ABR, and ECoG offer objective cochlear and auditory nerve data and are less challenging to perform.

Despite the significance of decreased ST in tinnitus patients, research exploring differences in auditory evoked tests based on ST status is relatively scarce. This study aims to investigate the features of auditory function alterations in tinnitus patients with reduced ST and to identify potential clues for the development of individualized treatment strategies. Our findings provide insights into the multifaceted auditory abnormalities linked to tinnitus and decreased sound tolerance, underlining the potential relevance of both peripheral and central auditory processes.

## 2. Materials and methods

### 2.1. Patient and data inquiry

We reviewed medical records from patients with subjective tinnitus who attended our tertiary university hospital's tinnitus clinic and reported tinnitus symptoms between March 2020 and June 2023. We set the exclusion criteria as: (1) pulsatile tinnitus in sync with heartbeat, (2) no conducted LDL tests, and (3) incomplete questionnaires, and (4) a history of previous ear surgery.

We collected data on age, sex, concomitant symptoms such as aural fullness, dizziness, headache, attention problems, temporomandibular joint (TMJ) discomfort, sleep disturbance, noise exposure or head trauma history, coexisting conditions like diabetes mellitus (DM), and hypertension (HTN). To assess attention problems, we asked patients if they had trouble concentrating due to tinnitus. Moreover, we conducted assessments for temporomandibular joint (TMJ) disorders. During patient interviews, we specifically inquired about any prior TMJ diagnoses. To ensure accuracy, we performed manual physical examinations of the temporomandibular area. We also documented the results of pure-tone audiometry, speech audiometry, ECoG, DPOAE, and ABR tests regarding audiological profiles. Psychoacoustic testing covered tinnitus characteristics, including laterality, character, pitch, loudness, minimal masking level (MML), and residual inhibition (RI). For the evaluation of RI, we added 10 dB to the MML. Patients were then instructed to listen to the RI stimulus for 1 min and subsequently asked to assess any changes in their tinnitus, categorizing it as partial suppression, complete suppression, or no suppression. Questionnaire responses were collected, including scores from the Tinnitus Handicap Inventory (THI), Beck Depression Inventory (BDI), and numerical rating scale (NRS) ratings regarding tinnitus awareness, annoyance, loudness, and its impact on daily life (6). We diagnosed decreased ST when the average of LDL measurements at 0.25, 0.5, 1, 2, 4, and 8 kHz was 77 dB or lower, categorizing patients into group 1. Patients above this threshold were considered normal ST and classified as group 2.

### 2.2. Statistical analysis

Statistical analysis was performed using IBM SPSS Statistics for Macintosh, version 29.0 (IBM Corp., Armonk, NY, USA), with *p-*values < 0.05 considered statistically significant. We calculated descriptive statistics for continuous variables such as age, tinnitus duration, pure-tone thresholds, tinnitus pitch, loudness, initial questionnaire scores, and NRS score, expressed as mean values (±standard deviation). The chi-square test compared categorical variables (sex, laterality, subjective symptoms) between groups, while the independent *t*-test contrasted continuous variables (ABR wave V threshold, latency, DPOAE responses) between groups. Receiver operating characteristic (ROC) curve analysis was applied to assess the diagnostic precision of DPOAE and ABR measurements in distinguishing between groups. Binary logistic regression analysis with the forward conditional method was implemented to discern independent associations between group differences, ABR, and DPOAE response at a specific frequency.

## 3. Results

### 3.1. Patient characteristics

This study incorporated data from 434 patients, with their specific characteristics outlined in Table 1. The patient cohort consisted of 213 men (49.1%) and 221 women (50.9%), and the mean age was 52.24 ± 14.77 years (range: 14–83). The average tinnitus duration spanned 28.05 ± 60.69 months. Tinnitus laterality was categorized unilateral in 205 patients (47.2%), bilateral in 180 patients (41.5%), and non-lateralized in 49 patients (11.3%), which refers to cases in which patients perceive tinnitus somewhere within their heads but do not localize it specifically to one or both ears. The mean pure-tone thresholds for the right and left ears were 19.63 ± 15.53 dB and 19.05 ± 13.29 dB, respectively. On the right side, the mean tinnitus pitch was 4.32 ± 10.55 kHz, and loudness was 7.79 ± 9.18 dB SL. Conversely, the left side displayed a mean tinnitus pitch of 3.86 ± 3.48 kHz and loudness of 6.87 ± 11.25 dB SL. Initial THI and BDI questionnaire scores were 46.50 ± 24.65 and 10.87 ± 8.79, respectively. The preliminary NRS scores for awareness, annoyance, tinnitus loudness, and tinnitus effect were 7.40 ± 3.02, 6.68 ± 2.78, 6.36 ± 2.33, and 5.21 ± 2.66, respectively.

**Table 1:** Patient characteristics.

**Variables**	**Total**	**Group 1**	**Group 2**	**P value**
Number	434	115	319	-
Age (years)	52.2 ±14.8	53.5 ± 14.8	51.8 ± 14.7	0.297
Sex				0.595
Male *n* (%)	213 (49.1)	54 (47.0)	159 (49.8)	
Female *n* (%)	221 (50.9)	61(53.0)	160 (50.2)	
Onset of tinnitus (months)	28.1 ± 60.7	29.6 ± 63.9	27.4 ± 59.5	0.743
Laterality				0.099^*^
Unilateral *n* (%)	203 (46.5)	45 (39.1)	160 (50.2)	
Bilateral *n* (%)	181 (42.4)	57 (49.6)	123 (38.6)	
Non-lateralized *n* (%)	43 (10.1)	13 (11.3)	36 (11.3)	
**Accompanying diseases**				
Diabetes mellitus *n* (%)	37 (4.7)	12 (11.7)	25 (13.0)	0.735
Hypertension *n* (%)	84 (10.6)	23 (22.3)	61 (30.2)	0.146
**Accompanying symptoms**				
Aural fullness *n* (%)	127 (16.8)	40 (42.6)	87 (44.6)	0.741
Subjective hyperacusis *n* (%)	88 (11.1)	31 (43.7)	57 (29.7)	0.033
Sleep disturbance *n* (%)	114 (14.4)	35 (67.3)	79 (53.3)	0.073
Headache *n* (%)	110 (13.9)	37 (34.3)	73 (30.9)	0.539
Temporomandibular/ neck pain *n* (%)	61 (7.7)	22 (52.4)	39 (37.5)	0.099
Trouble focusing *n* (%)	34 (4.3)	10 (37.0)	24 (34.8)	0.835
Dizziness *n* (%)	78 (9.9)	26 (25.5)	52 (24.4)	0.836
**Hearing thresholds (dB)**				
Right	19.6 ± 15.5	21.1 ± 16.3	19.1 ± 15.2	0.251
Left	19.0 ± 13.3	19.1 ± 12.7	19.0 ± 13.5	0.99
**Initial questionnaires**				
THI	46.5 ± 24.7	50.0 ± 24.0	45.3 ± 24.8	0.09
BDI	10.9 ± 8.8	12.0 ± 9.4	12.0 ± 9.4	0.107
**Numerical rating scale (0–10)**				
Awareness	7.4 ± 3.0	7.8 ± 2.9	7.3 ± 3.1	0.189
Annoyance	6.7 ± 2.8	6.8 ± 2.8	6.6 ± 2.8	0.631
Loudness	6.4 ± 2.3	6.6 ± 2.3	6.3 ± 2.3	0.134
Effect on life	5.2 ± 2.7	5.6 ± 2.8	5.1 ± 2.6	0.123

Data are presented as mean ± standard deviation for numerical variables and number (%) for nominal variables. THI; Tinnitus handicap inventory, BDI; Beck depression inventory. ^*^Fisher's exact test.

### 3.2. Audiological and DPOAE findings: group comparison

No significant differences between the two groups were observed regarding parameters such as age, onset, sex, laterality, and subjective symptoms like sleep disturbance, headache, temporomandibular/neck pain, trouble focusing, dizziness, aural fullness, and comorbidity with diabetes or hypertension except subjective hyperacusis (*p* > 0.05). Similarly, there were no significant disparities between the two groups in THI, BDI, and VAS for tinnitus awareness, annoyance, loudness, and effect (*p* > 0.05).

Audiological tests, including the mean PTA and SP/AP ratio, failed to demonstrate any significant differences between the two groups (*p* > 0.05). However, a significant difference in the ABR wave V threshold for both sides between the groups was evident (*p* = 0.028 for the right side, *p* = 0.002 for the left side). Group 1 exhibited lower values (Right side: Group 1: 30.27 ± 14.41 dBnHL, Group 2: 33.93 ± 16.63 dBnHL; Left side: Group 1: 30.27 ± 12.66 dBnHL, Group 2: 34.87 ± 14.80 dBnHL). Moreover, Group 1 demonstrated significantly reduced latency of ABR wave V for both sides (*p* = 0.017 for the right side, *p* < 0.001 for the left side). However, there was no significant deviation in the V/I amplitude ratio and latency of wave I, III, and I-III between the groups (*p* > 0.05).

**Figure 1:**
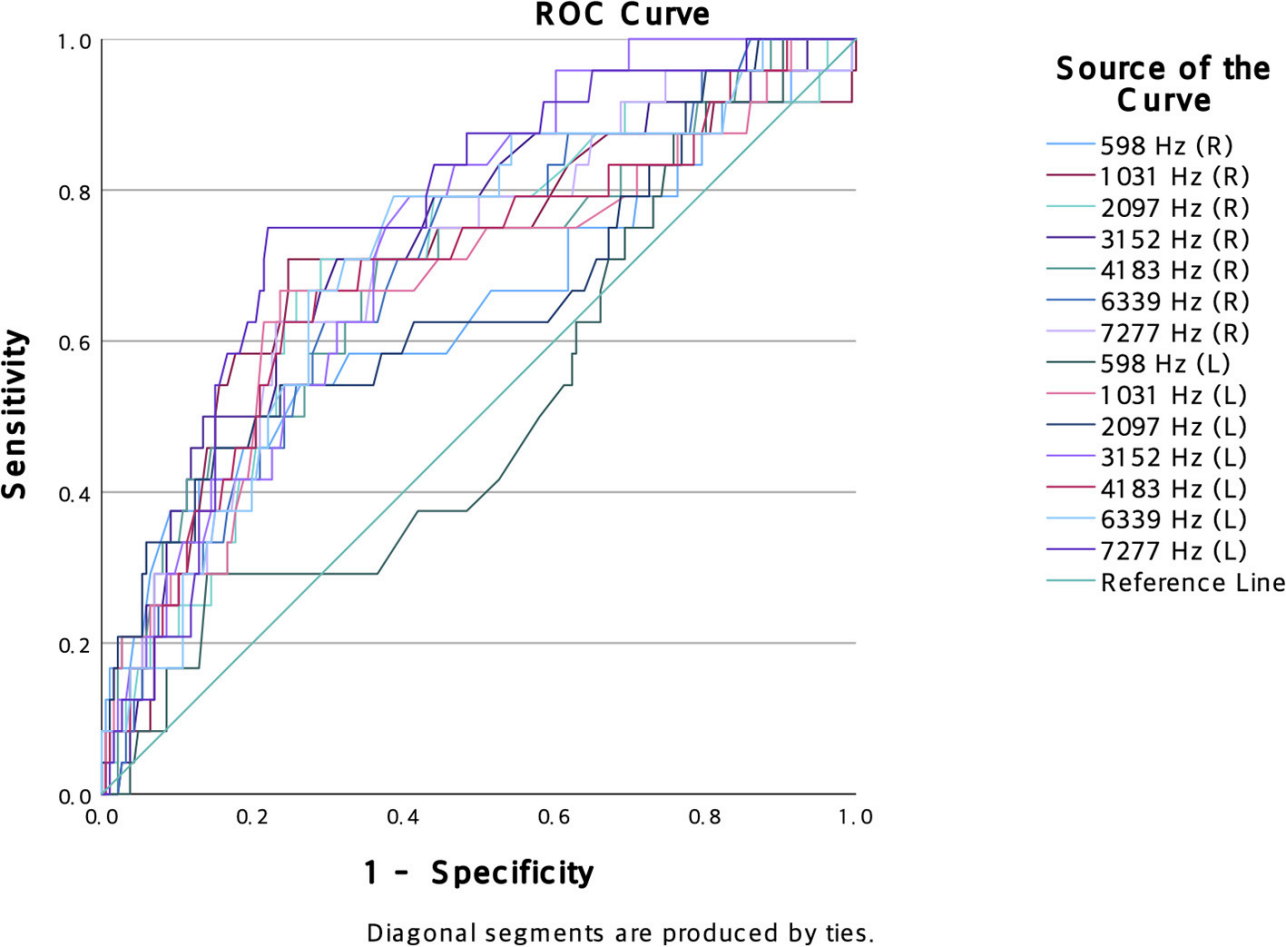
Results of ROC curve analysis on DPOAE. Illustrates the results of ROC curve analysis, which assesses the diagnostic capability of various frequency measurements in distinguishing between two groups: Group 1 and Group 2. The curves represent various frequencies, with each curve's AUC indicating its discriminatory power. The higher the AUC, the better the frequency discriminates between the two groups.

With respect to DPOAE, all measured frequencies showed significant mean differences between the two groups (*p* < 0.01). Group 1 demonstrated notably increased DPOAE responses compared to Group 2 at all frequencies. ROC curve analysis indicated that the majority of DPOAE measurements could distinguish between the groups, except at the 598 Hz frequency on the left side (Figure 1, Table 2). Crucially, 7,277 Hz in the left ear showed the best discriminatory power. This was confirmed by a binary logistic regression analysis, which employed the forward conditional method and demonstrated a statistically significant relationship between group membership and the DPOAE response at 7,277 Hz in the left ear [B = 0.093, *p* < 0.001, EXP(B) = 1.07, 95% CI = 1.044–1.153]. These results indicate that while DPOAE responses exhibited significant differences across all tested frequencies between Group 1 and Group 2, the frequency of 7,277 Hz in the left ear stands out as a particularly noteworthy discriminator of group variance, highlighting its potential as a key biomarker for identifying individuals with tinnitus and decreased sound tolerance.

**Table 2:** Results of ROC curve analysis on DPOAE.

	**Frequency**	**Cut-off value**	**AUC**	**S.E**.	**P value**	**95% Confidence interval**	

						**Lower**	**Upper**
Right	598 Hz	8.250	0.640	0.069	0.026	0.503	0.776
	1,031 Hz	10.150	0.702	0.064	0.001	0.577	0.827
	2,097 Hz	9.400	0.695	0.059	0.002	0.580	0.810
	3,152 Hz	10.050	0.725	0.057	0.000	0.613	0.836
	4,183 Hz	10.050	0.687	0.062	0.003	0.566	0.809
	6,339 Hz	9.800	0.692	0.055	0.002	0.584	0.801
	7,277 Hz	9.200	0.703	0.057	0.001	0.591	0.815
Left	598 Hz	9.500	0.510	0.062	0.878	0.388	0.631
	1,031 Hz	9.800	0.678	0.064	0.005	0.552	0.804
	2,097 Hz	13.150	0.651	0.066	0.016	0.521	0.781
	3,152 Hz	9.300	0.734	0.046	0.000	0.643	0.825
	4,183 Hz	13.250	0.691	0.061	0.002	0.571	0.810
	6,339 Hz	9.950	0.707	0.055	0.001	0.598	0.816
	7,277 Hz	8.150	0.762	0.048	0.000	0.667	0.856

AUC, area under the curve; S.E., standard error.

## 4. Discussion

In this study, an analysis of data from 434 patients was conducted. The amplitudes of DPOAE revealed significant mean variations, with Group 1 demonstrating enhanced responses relative to Group 2 across all frequency ranges. Moreover, an increased DPOAE response at 7,277 Hz in the left ear was identified as an independent risk factor for tinnitus associated with diminished ST, suggesting that DPOAEs hold potential as objective biomarkers for decreased sound tolerance in individuals with tinnitus. Notably, ABR wave V threshold and latency also displayed significant discrepancies, indicative of intergroup auditory processing variations.

Based on the ROC curve analysis and binary logistic regression analysis, the increased DPOAE response at 7,277 Hz in the left ear has strong potential as a biomarker for identifying individuals with tinnitus and decreased ST. That is, measuring DPOAE responses may provide additional information to predict the occurrence of tinnitus and decreased ST better than ABR or patient characteristics. Most of the higher AUC values for DPOAE response than 0.5 indicated that DPOAE measurement effectively distinguishes between individuals with tinnitus and decreased sound tolerance (Group 1) and those without (Group 2). The left ear specificity may result from the functional asymmetry of the brain because the right hemisphere processes emotion, spatial awareness, and music (8, 9). In contrast, the left hemisphere processes language and speech but requires further validation (8–10).

Initially, we established the criterion for decreased ST using the LDL test, setting a specific cutoff value at 77 dB. This choice was based on prior research (7) and the correlation with subjective hyperacusis, as indicated in our previous studies (manuscript currently under revision in other journals). This established benchmark allowed us to identify patients exhibiting increased auditory sensitivity, providing a more quantifiable metric for our analysis.

Subsequently, heightened DPOAE responses in Group 1 relative to those without impaired ST across all frequencies suggest a potential association between sound hypersensitivity and modified functionality of the cochlea's outer hair cells (OHCs). These observations align with earlier studies contrasting DPOAEs between tinnitus sufferers with normal hearing and control groups, especially in cases of hyperacusis, where elevated amplitudes within the 1,501–5,005 Hz frequency range were reported (8). These studies postulated that the escalated DPOAE response could be attributed to enhanced OHC motility prompted by diminished activity of the olivocochlear efferent fibers (11, 12). In addition, these studies revealed that decreased DPOAE amplitudes were particularly exhibited at 2,002 Hz in the presence of misophonia, a finding contrasting with hyperacusis (11). In addition, these findings align with a UK population-based study that reported 3.7% of children experiencing hyperacusis, with a similar link to increased DPOAE responses but no other auditory factors (13).

Contrary to lateral olivocochlear efferents that target auditory nerve fibers, medial olivocochlear (MOC) efferents originating from the medial nucleus of the trapezoid body (MNTB) within the superior olivary nucleus, innervate OHCs and have the potential to mitigate acoustic trauma damage by dampening cochlear amplification (14). Additionally, selective attention can modulate MOC effects. In animal studies, irrelevant auditory stimuli were suppressed during selective visual or olfactory stimulation, a process associated with MOC efferents (15). Therefore, we speculate that dysfunctional MOC efferents may contribute to decreased ST in patients, causing less suppression of irrelevant sounds. This effect might not depend on hearing status as we observed the same in tinnitus patients without any hearing restrictions, contradicting the findings of a previous study (11).

Furthermore, MOC efferents regulate OHCs through acetylcholine release, thus suggesting that acetylcholine administration might benefit tinnitus patients with reduced ST. In the context of myasthenia gravis, a condition associated with acetylcholine receptor autoantibodies, the administration of acetylcholine (60 mg of pyridostigmine bromide) modified DPOAE amplitudes, particularly in middle to high frequencies (16). Correspondingly, our discovery of a unique association between group differences and DPOAE response at 7,277 Hz in the left ear indicates that DPOAE responses at this specific frequency might serve as potential biomarkers for differentiating tinnitus patients with or without decreased ST or for assessing treatment outcomes. In general, acetylcholinesterase inhibitors such as pyridostigmine are administered through intravenous administration or oral medication. Previously known side effects range from flu-like symptoms, hot flashes, and increased salivation to increased bronchial secretion, irregular heartbeat, and chest pain, posing potential risks (17). Additional research on local treatments, such as intratympanic injection, is needed to mitigate systemic side effects. However, developing a method for administering drugs to target the nicotinic or muscarinic acetylcholine receptors in the inner ear remains a challenge that necessitates further investigation (18). Meanwhile, in addition to acetylcholine, existing pharmacologic treatments used to address decreased ST include bisphosphonate risedronate, fluvoxamine, fluoxetine, gabapentin, clonazepam, and carbamazepine (19).

Conversely, the clinical relevance of DPOAE in tinnitus or hyperacusis is contested by certain investigators. A subset of studies propose that the DPOAE response exhibits no variance based on the presence or duration of tinnitus or hyperacusis (20, 21). Rather, the evidence points toward extended high-frequency (EHF) hearing loss being a more impactful determinant than DPOAE amplitudes or I/O functions in normoacoustic adults (20). Moreover, adolescents with chronic tinnitus were found to have a significant decline in LDL in comparison to counterparts without tinnitus or with intermittent tinnitus (21). These diverse results emphasize the complex nature of decreased sound tolerance and underscore the importance of considering both peripheral and central mechanisms. While our study offers valuable insights into the potential of high-frequency DPOAEs as biomarkers, it also highlights the necessity of a comprehensive approach, including factors like EHF thresholds and central auditory processing, for a thorough understanding of conditions like tinnitus and hyperacusis, ultimately informing future research and clinical practices.

Another key finding in our study is the shortened latencies in ABR wave V among tinnitus patients exhibiting decreased ST. These shortened latencies shed light on altered auditory processes, suggesting a systemic state of hyperexcitability within this particular subgroup. In alignment with our observations, a study involving children with autism and auditory hypersensitivity displayed shortened ABR wave V latencies in comparison to the control group (22). Furthermore, shorter ABR interpeak latencies I-V and III-V were discovered in the autistic group vs. the control group. However, a significant proportion of tinnitus investigations disregard auditory hypersensitivity or hyperacusis, despite approximately two-thirds of tinnitus patients possibly having a decrease in LDL (i.e., lower dB) and inconsistent reports of changes in ABR wave V. For instance, elongation of ABR wave V latency has been reported to correlate with the transition from intermittent to persistent tinnitus (23). In the context of noise-induced cochlear synaptopathy, a rise in ABR wave V latency commensurate with increasing background noise level was noted, signifying disruption in the auditory nerve response and temporal processing (24). It was surmised that modifications in ABR wave V latency were primary driven by alterations in the auditory nerve response as opposed to cochlear excitation levels in cochlear synaptopathy.

Regarding the observation of diminished ABR V amplitude, this deviates from the typical findings associated with cochlear synaptopathy. One could postulate that auditory deafferentation due to cochlear synaptopathy may precipitate compensatory alterations in the central auditory pathway, resulting in phenomena such as hyperacusis or amplified central gain (25, 26). Decreased ABR I amplitude and enhanced ABR V/I amplitude have been identified as characteristic findings that substantiate these phenomena. Such outcomes have been a subject of investigation in human studies, mirroring trends observed in animal research. Our prior study also documented an augmented III/I and V/I ratio in bilateral tinnitus patients compared to a normal control group, suggesting a correlation between cochlear nucleus level hyperexcitability and bilateral tinnitus (27). In congruence with these findings, acute tinnitus patients displayed a decreased ABR I amplitude and an increased ABR V/I amplitude, exhibiting a correlation with age and pure-tone thresholds (28). These patterns tend to be more salient when factors such as high-frequency hearing loss, age, and gender are more meticulously controlled (29).

If the principal lesion were located at the anatomical site correlating to the increased DPOAE response, a parallel change would be expected in ABR wave I. However, given the absence of variation in all other results, with the exception of the threshold and latency of ABR wave V, it suggests the possibility of pathological alterations confined to the region of ABR wave V's origin. The generation of ABR wave V is attributed to the medial superior olivary complex, which projects extensively to the lateral lemniscus and a fraction of the inferior colliculus (30). Furthermore, considering the MOC efferents commence from the superior olivary complex and inhibit outer hair cells, the primary lesion is plausibly located either at the superior olivary complex or along the central auditory pathway beyond the SOC. This hypothesis necessitates validation through subsequent radiological research. Corroborating this, studies on thalidomide-induced autism in rats demonstrate a reduction in calbindin d28k immunoreactivity within the SOC, along with a significant decrease in MNTB thickness compared to the control group. This implies that auditory hypersensitivity might stem from impaired inhibitory processing within the auditory brain center (31). A range of pharmacological interventions, encompassing selective serotonin reuptake inhibitors and atypical antipsychotics such as risperidone, aripiprazole, and N-acetylcysteine, are employed for autism treatment. These medications, aimed at managing irritability and restoring the balance between excitation and inhibition, show potential for application in the treatment of reduced SL, a prospect warranting further exploration (32).

Lastly, no significant differences were identified between the two groups in terms of most demographic characteristics, subjective symptoms, or questionnaire scores related to tinnitus (*p* > 0.05). It is acknowledged that hyperacusis risk factors include hearing loss, female gender, certain medical conditions such as Williams syndrome and autism, specific occupations like musicians and teachers, lower income, tinnitus, and both physical and mental health complications (6). Our previous study involving 194 tinnitus patients showed that a younger age, heightened THI and BDI scores, increased NRS score for tinnitus awareness, and impact on life were observed in patients diagnosed with both tinnitus and hyperacusis (6). The discrepancy between this study and our preceding work can be attributed to the stringent criterion for decreased discomfort levels (ST), set at a mean LDL value of up to 77 dB in the current study. LDL testing, while essential, can instigate discomfort in patients and necessitates their cooperation. Consequently, incorporating an LDL test for all tinnitus patients poses a diagnostic challenge for decreased ST. Moreover, varying interpretations of hyperacusis using LDL among researchers may influence the findings of this study.

This study is not without limitations. The study's retrospective design and the inherent challenge of conducting LDL testing on all tinnitus patients may have imposed some bias on the results. Furthermore, inconsistent definitions of hyperacusis, as based on LDL, across various research initiatives could have potentially affected the interpretation of our results (6). The European School for Interdisciplinary Tinnitus Research-Screening Questionnaire (ESIT-SQ) is a recently developed questionnaire that facilitates the standardized clinical profiling of tinnitus-related data and encompasses various questionnaires beyond the items investigated in this study (33). If ESIT-SQ had been additionally employed in this study, it would have provided a more comprehensive understanding of the characteristics of the groups that could potentially impact the study's results. For instance, we found that the enrolled patients in this study reported fewer instances of aural fullness or headaches than the ESIT-SQ data from Ménière's disease patients (34). Given that ESIT-SQ includes a more diverse range of evaluation items, its active use in future research may prove more effective in comparing research outcomes.

## 5. Conclusion

Our study elucidates the auditory characteristics of tinnitus patients with reduced ST, demonstrating an enhanced DPOAE response in tandem with a shortened ABR wave V latency and attenuated amplitude. Such discoveries could potentially imply the involvement of the central auditory pathway, specifically at or beyond the superior olivary complex, in the expression of tinnitus and diminished sound tolerance. The amalgamation of DPOAE measurements and ABR analysis provides valuable insights into the integral auditory pathophysiology. Future research is warranted to substantiate these findings with radiologic or anatomical evidence, elucidate the precise mechanisms that connect these auditory anomalies, and examine their clinical implications.

## Data availability statement

The raw data supporting the conclusions of this article will be made available by the authors, without undue reservation.

## Ethics statement

The studies involving humans were approved by the Institutional Review Board of the Ewha Mokdong Hospital. The studies were conducted in accordance with the local legislation and institutional requirements. The Ethics Committee/institutional review board waived the requirement of written informed consent for participation from the participants or the participants' legal guardians/next of kin because retrospective nature of the study.

## Author contributions

EK: Formal analysis, Writing—original draft, Writing—review & editing. S-HS: Writing—review & editing. SB: Writing—review & editing. HL: Writing—review & editing, Conceptualization, Methodology, Writing—original draft.
